# Nutritional and health status of a cohort of school-age children born to mothers treated for severe acute malnutrition in their childhood in The Democratic Republic of Congo

**DOI:** 10.1371/journal.pone.0269527

**Published:** 2022-06-07

**Authors:** Christine Chimanuka Murhima’Alika, Serge Balolebwami Zigabe, Yvette Bahati Lufungulo, Pacifique Mwene-Batu Lyabayungu, Daniel Garhalangwa Mayeri, Amani Ngaboyeka Gaylord, Michèle Dramaix, Philippe Donnen, Ghislain Bisimwa Balaluka

**Affiliations:** 1 Ecole Régionale de Santé Publique, Université Catholique de Bukavu, Bukavu, Democratic Republic of Congo (DRC); 2 Centre de Recherche en Sciences Naturelles, Lwiro, Democratic Republic of Congo (DRC); 3 Hôpital Provincial General de Reference de Bukavu, Université Catholique de Bukavu, Bukavu, Democratic Republic of Congo (DRC); 4 Ecole de Santé Publique, Université Libre de Bruxelles, Brussels, Belgium; Public Library of Science, UNITED KINGDOM

## Abstract

**Background:**

Malnutrition is a public health problem, but outside the theoretical framework, little is known about the concrete intergenerational effects of malnutrition.

**Objective:**

The objective of this study is to compare the nutritional status and health indicators of school children born to mothers who were treated for severe acute malnutrition (SAM).

**Methodology:**

The study took place in Miti-Murhesa health zone in the Democratic Republic of Congo. This is a cohort study assessing the nutritional and health status of school children born to mothers who had been treated for SAM, based on WHZ or edema, in Lwiro hospital between 1988–2002 compared to children born to mothers who were not exposed to SAM. Stunting and thinness were evaluated by Height for Age Z-score (HAZ) and Body Mass Index by Age criteria (BMIAZ) respectively. On admission, blood samples were taken to assess anemia, HIV serology, hemogram and others biological indicators. Stool’s examinations were conducted by using Olympus optical microscope. Parametric and non-parametric tests were applied to compare the different variables in two groups.

**Results:**

We identified 106 children aged 5–16 years (103 exposed and 58 unexposed) and we received 83.5% and 91.4% children respectively for anthropometric parameters. The mean of age was 7.9 ± 2.4 year in exposed group and 7.4 ± 2.1 year in unexposed group (p = 0.26). The prevalence of stunting was 68.3% in the exposed group and 67.3% in the unexposed group (p = 0.90). The prevalence of thinness was 12.8% in the exposed group and 9.6% in the unexposed group (p = 0.57). The biological profile (glycemia, urea, creatinine and hemogram) and the prevalence of intestinal parasites were similar in the two groups.

**Conclusion:**

In this sample, in a malnutrition-endemic area, there was no statistically significant difference in nutrition and health indicators between school children born to mothers exposed to SAM and their community controls.

## Background

Malnutrition is a health problem throughout the world. According to the latest report the proportion of children aged 6–59 months who suffer from stunting is 22.2% corresponding to 150.8 million children while those suffering from wasting represent 7.5% corresponding to 50.3 million children [1, 2]. Malnutrition is one of the main causes of mortality in children under 5 years of age [3] and it is estimated that 45% of deaths of children under 5 years of age are attributable to malnutrition [4, 5].

In addition to the risk of mortality, malnutrition in childhood exposes survivors to other major risks in adulthood (metabolic diseases such as diabetes, obesity and hypertension). Currently, several research teams are working on the long-term effects of malnutrition with the objective of exploring the causal relationship between malnutrition in childhood and metabolic diseases in adulthood. Most of these studies focus on adults and adolescents who have directly experienced acute malnutrition during childhood [6–11].

However, little is known about the reproductive health of mothers who were malnourished in childhood and the condition of their offspring, even though it is generally known that maternal health, particularly maternal height, is an indicator of the intergenerational links between nutrition and maternal and child health [12].

To our knowledge there are no publications that trace the situation of school-age children born to mothers who suffered from SAM in childhood in sub-Saharan Africa region. It is very likely that our study is one of the first because we did not find another one in our documentation.

Malnutrition and intestinal parasitic infections are often considered as indicators of the health status of school-age children. The prevalence of undernutrition and parasitic infections varies greatly from one region to another, but more authors estimate that more than 50% of school children in sub-Saharan Africa are infected with soil-borne helminths [13]. However, the high presence of intestinal parasites is an indicator of the poor hygiene conditions generally observed in poor environments where there is a culture of open defecation and little access to routine deworming. Recent publications consider open defecation and lack of sanitation as a determinant of chronic malnutrition [14]. Although the challenge on the need to separate linear growth retardation and stunting, researchers agree that stunting is the consequence of repeated episodes of acute malnutrition and wasting or an organism adaptation of the growing child to this state of undernutrition [15]. Stunting is therefore a good indicator to assess the nutritional capital of the child and the environment in which it evolves. Also, in the absence of a standard definition of "acute malnutrition" in children over 5 years of age, the body mass index for age (BMIAZ) indicator is used to assess thinness. To strengthen the prevention of malnutrition around the world, the Global Multisectoral Movement on Nutrition (Scaling Up Nutrition—SUN), had proposed to expand nutritional surveillance beyond children under 5 years of age to include other specific groups such as children 13–15 years and women 15–49 years of age [16].

The Assessment of child health indicators related to stunting determinants can help understand the level of vulnerability of specific groups in the context of endemic malnutrition [14]. The main objective of this study is to assess the nutritional status of school-aged children born to mothers who were exposed to severe acute malnutrition (SAM) during childhood and secondarily to describe the profile of child health indicators observed in these children when they live in a nutritionally vulnerable environment. We sought to compare the nutritional status and overall health of children born to mothers exposed to MAS with other children in the same community.

## Material and methods

### Study region

The study took place in the health zone (HZ) of Miti Murhesa located in the South Kivu province in the east of the Democratic Republic of Congo (DRC), 33 km from the city of Bukavu. In this health zone is the Natural Sciences Research Center of Lwiro (CRSN) where the Lwiro Pediatric Hospital (HPL) is located. The HPL was used as the provincial reference hospital for acute malnutrition from 1980 to 2003 before the full integration of malnutrition management in the different hospitals in all health zones in accordance with the country’s health policy [17, 18]. South Kivu province has had high rates of malnutrition for the past two decades [19–21] and malnutrition is endemic in the Miti-Murhesa health zone [22, 23]. It is a region located at altitudes of 1500 and 2500 m (Katana on the shores of Lake Kivu and Miti-Murhesa along the Kahuzi-Biega Park, world-renowned for the mountain gorilla species it shelters). The population lives essentially from self-subsistence agriculture with traditional food practices in a context of poverty and overpopulation [23–25]. A recent study traced the cohort of former malnourished people treated at HPL between 1988–2007, some of whom already have school-age children [26].

### Conception and study design

It is a study designed to investigate the intergenerational effects of SAM in an endemic malnourished environment. We conducted the study to assess the nutritional status and some nutritional and biological indicators of school-aged children in a cohort of children born to mothers who were exposed to SAM in childhood [26]. The community controls are children of the same age born to mothers living in the same villages but who were not exposed to SAM in childhood (no reported admissions to a nutritional center during childhood according to health workers and the different registers of the Lwiro Hospital and nutritional centers in the Miti-Murhesa (HZ). Data collection was conducted from March to May 2019.

### Study subjects and identification

For each mother identified in the Lwiro cohort (exposed to SAM), we recorded her child aged 5–16 years when she was in the "exposed" group. Community controls of the same age were then recruited from the closest households in the same village whose mothers had not been exposed to SAM as their childhood. To identify these households, Community Health Workers (CHWs) spun a bottle in the home of the case, then CHWs searched door-to-door, starting from the house closest to the direction indicated by the bottle until they found a household where the mother had no history of SAM during her childhood. The research team cross-referenced information from the nutritional centers, CHW and chiefs of villages to ensure that the mothers of children included in the unexposed group had never suffered from SAM in childhood. Recruitment was conducted by a multidisciplinary research team (doctors, nurses, nutritionists, laboratory technicians, and CHW), some members had collaborated with LPH in the implementation of a community-based nutrition program between 1994–2002. More than half of the CHWs were familiar with the households that had malnourished children in the different villages [27].

### Outcomes

The main expected outcome is to compare the prevalence of stunting and thinness in the two groups of children (exposed and non-exposed). For this purpose, our study aims to evaluate the prevalence of stunting according the Height for Age Z-score (HAZ) and the prevalence of thinness was measured according to the Body Mass Index by Age criteria (BMIAZ). The secondary objective was to compare the some nutritional and health indicators commonly assessed in the child health program: thinness, anemia, C-reactive protein (CRP), the human immunodeficiency virus infection (HIV), glycemia, creatinine, urea, hemogram, intestinal parasites).

### Sample size

Given the insufficient number of children born to mothers exposed to SAM the sample size was limited to the number of children aged 5–16 years born to mothers with a history of SAM in childhood that could be identified in the Lwiro cohort. These exposed children were matched to community controls on a 1:1 basis. However, to better detect the 15% difference in stunting prevalence between the two study groups with a statistical power of 80% and an alpha error of 0.05 we needed a minimum of 209 children per group [28]. This should correspond to 35% in unexposed group and 50% in exposed group with reference to the prevalence of stunting observed in areas of high vulnerability in Eastern DRC [22]. Nevertheless, the research team enrolled all children in the Health Zone whose mothers had been identified as having been exposed to SAM in their childhood. We identified a total of 103 children aged 5–16 years in the cohort but 86 were presented by their mothers for anthropometric and other tests. We also recruited 58 non-exposed children but 53 had completed the appointment.

### Anthropometric parameters measurement

The body weight was measured to the nearest 100 g using an electronic scale (OMRON, HN-289-EBK), the subject being dressed only in light clothing. The standing height was determined with a SECA 206 cm^®^ mesh in a subject without shoes to within 0.1 cm. The Mid-Upper-Arm Circumference (MUAC) was taken with the Kashir strip. Anthropometric measurements were performed in accordance with WHO guidelines [29, 30] and were subject to quality control involving independent taking by two team members. The final measurement was the average of the two. In case of a difference of more than 200 g for weight and 0.5 cm for standing height a third measurement was taken. The average of the two closest measurements was used. Edema was considered to be permanent bilateral edema on the day with positive cup sign.

The tricipital skin fold was measured with the Harpenden’s Compass in children who were undressed, standing or sitting with the arm along the body. Using a tape measure, the mid-distance between the acromion and the olecranon was marked with a pen. Then we firmly and vertically grasped between thumb and index finger the skin fold on the posterior side of the arm at the height of the pen line, taking care to include the subcutaneous tissue and exclude the underlying muscle tissue. With the other hand we measured the thickness of the skin fold with the help of the measuring device, making sure that it was zero. Finally, the jaws of the forceps were released to exert a constant pressure on both sides of the skin fold and waited for 3 seconds. Body mass index (BMI) was calculated as weight/height^2^. Stunting was assessed according to the Height-for-Age Z-score (HAZ) according to the WHO references for children aged 5–19 years [31]. We assessed thinness according to the Body Mass Index -for-Age indicator (BMIAZ) by using the curve for children aged 5–19 years using the -2 threshold [32].

For the Z-scores, we considered values ranging from -5.9 to +5.9.

### Blood sampling, hemoglobin measurement and HIV serology

For convenience and taking into account the logistical limitations in organizing the collection of blood samples and laboratory analyses, we have planned biological samples for a minimum of 50% of children per group except for HIV serology and hemoglobin examination (random selection based on order of arrival at the health center). The blood samples were taken with the parents’ agreement, who then agreed to bring the children to one of the targeted health centers during the week of data collection (Kavumu, Lwiro, Buhandahanda, Chegera, Kabushwa, Mulungu).

A capillary blood sample was taken to measure haemoglobin using the Hemocue^®^ Hb 201+ devices [33, 34]. The child was classified as anemic if haemoglobin < 12.0 g/dL. The HIV serology test was performed using the Determine ALERETM HIV COMBO test [35]. Venous sampling was performed by appointment for the determination of blood count, blood glucose, creatinine and urea [36, 37]. The Mean Corpuscular Volume (MCV), Mean Corpuscular Haemoglobin (MCH) and Mean Corpuscular Haemoglobin Concentration (MCHC) were measured. Blood numeration was done using a Coulter Counter SYSMEX XP-300. The determination of blood urea was carried out by the Berthelot method and the determination of creatinine by the Jaffe method with reagents from Cypress Diagnostic. We used the Automatic Biochemistry Analyzer Rayto chemray 120 manufactured in China (Shenzhen) [36–38].

The C-reactive protein was firstly investigated qualitatively with the Cypress Rapid Kit and then measured quantitatively with the PCR automat (GENRUI PA-120). The HIV test and the blood count were offered to all subjects included in the study, while the measurement of glycemia, urea and creatinine was limited to one subject out of two in each group. For subjects in whom creatinine was measured, creatinine clearance or glomerular filtration rate (GFR) was calculated by the Schwartz formula (Cl = (K*Height in cm) / creatinine in mg/dL); K being a parameter that varies with age; and we considered the threshold of GFR = < 110 mL/min to define normal state [39].

### Sampling and direct stool examination

One to two consecutive samples of fresh stool were collected from children (exposed and unexposed) whose mothers agreed to participate in this test because the stool had to be collected at the health center. Fresh stool was collected in a sterile jar given to the mother, and once the stool was collected, the jar was collected by the interviewer and given to the laboratory technician within 30 minutes of defecation. Direct examination of stools was performed after mixing with a plastic rod of at least 2mg of stools diluted with saline (when they had a solid consistency) on a slide with an object. After covering this sample with a slide, it was examined on the Olympus optical microscope (Model CX21iFS1-SN18M0043) using first the 10X objective and then 40X systematically [40]. Following the same method, a second slide was stained with lugol to allow the identification of cysts.

### Other variables for children

The child’s age was checked in the health record before confirming the information received from the mother. The child was questioned about his level of schooling and the name of his school before this information was confirmed by the mother or accompanying person. Schooling was categorized into three categories: (i) not attending, (ii) optimal schooling when the child was not yet behind in school relative to his or her age, and (iii) delayed schooling when the child was at least one grade behind in school relative to his or her age. This delay may be due either to late enrollment or to repetition in any class.

#### Variables for mothers

We recorded their age, marital status, and parity. Their physiological status was categorized into four categories (pregnant women, lactating mother, pregnant and lactating mother, non-pregnant and non-lactating mother). Body weight was measured to the nearest 100 g using an electronic scale (OMRON, HN-289-EBK) while the subject was dressed only in light clothing. Height was determined to the nearest 0.1 cm without shoes using a SECA 206 cm^®^ measuring tape attached to a wall. The MUAC was measured using an adult MUAC tape.

### Statistical analysis

The data was encoded on Access and then analyzed on SPSS 23. The quantitative variables were presented as means and standard deviations (SD) or medians and minimum-maximum (min-max) depending on whether the distribution was symmetrical or not. Categorical variables were summarized as frequency and proportion. Pearson’s Chi^2^ test or Fisher’s exact test if the Chi^2^ was not valid was used to compare the distributions of categorical variables in the two groups (exposed and unexposed). The student t-test was used to compare the means between the two groups. The Mann Whitney test was applied to compare the medians between the two groups when the distribution was skewed.

### Ethics approval and consent to participate

The study protocol was approved by the Ethics Committee of the Catholic University of Bukavu under reference UCB/CIES/NC/02B/2019 after obtaining authorization from the Provincial Health Division of South Kivu (ref. 004/CD/DPS-SK/2016). All mothers whose children participated in the study gave written informed consent.

## Results

**Fig 1:**
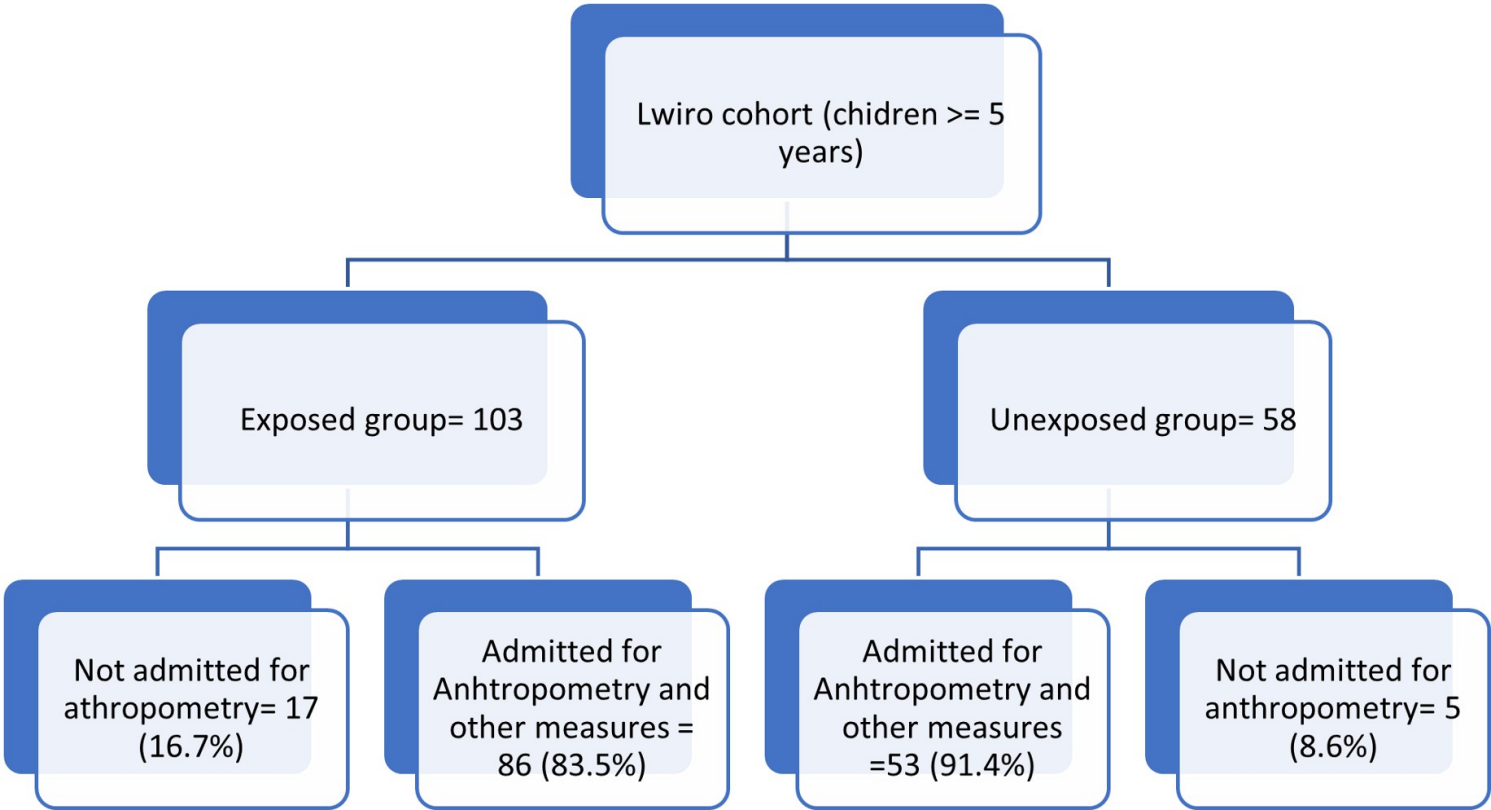
The study flow chart.

Fig 1 shows the flow chart of the study: the subjects identified in the cohort and those who were included in the study. We identified 103 exposed and 58 unexposed children. For the anthropometric parameters, we received 86 exposed and 53 unexposed children respectively 83.5% and 91.4% participation rate.

Table 1 shows that the mean of age was 7.9 ± 2.4 year in exposed group and 7.4 ± 2.1 year in unexposed group (p = 0.26). The percent of children under 12 years was 92.2% in the exposed group and 94.8% in unexposed group (p = 0.75). The proportion of girls was 58.3% and 55.2% in the exposed and unexposed groups respectively (p = 0.70). The proportion of children not attending school was 29.5% for the exposed and 30.2% for the unexposed (p = 0.86). The average age of the mothers was similar in both groups and the majority were lactating mothers. The percentage of mothers with a height of less than 150 cm was 35.9% in the exposed group and 29.3% in the unexposed (p = 0.39). The mean of mother’s MUAC is higher in the unexposed group with a statistically significant difference. The percentage of mothers with a MUAC of less than 230 mm was 12.4% in the exposed group and 3.1% in the unexposed (p = 0.07).

**Table 1:** Socio-demographic characteristics.

Variables	Mother exposed to SAM		*Mother no exposed to SAM*		*p*
	n	Mean ± DS or %	n	Mean ± DS or %	
***Children***					
**Age (years)**	103		58		
Mean (SD)		7.9 ± 2.4		7.4 ± 2.1	0.26
5–11 years	95	92.2%	55	94.8	0.75*
12–16 years	8	7.8%	3	5.2	
**Sex**					
Female	60	58.3%	32	55.2%	0.70
Male	43	41.7%	26	44.8%	
**Rank** Med (Min-Max)	88	2 (1–6)	51	3 (1–8)	0.001
**Scholarization**	88		53		
Not attending	26	29.5%	16	30.2%	0.86
Deleyed schooling	37	42.5%	20	37.7%	
Optimal schooling	25	28.4%	17	32.1%	
***Mothers***					
Age (years)	93	28.1± 4.5	50	28.1± 5.6	0.97
Height (cm)	103	150.7± 8.1	58	152.3 ± 4.8	0.16
Height < 150 cm	103	35.9%	58	29.3%	0.39
Height < 145 cm	103	18.4%	58	5.2%	0.02
Weight (Kg)	103	51.6 ± 8.9	58	55.1 ± 7.2	0.01
MUAC (mm)	103	245.6 ± 33.9		261.9 ± 26.4	0.002
MUAC < 230 mm	103	12.4%	58	3.1%	0.07
Physiological status	103		58		
Lactating mothers	55	53.4%	39	67.2%	0.08
Pregnant women	28	27.2%	6	10.3%	
Pregnant and lactating	5	4.9%	2	3.4%	
Not pregnant- Not lactating	15	14.6%	11	19.0%	

(*) Fisher Exact Test. SAM Severe acute malnutrition in childhood

Table 2 shows that there was no difference between the exposed and unexposed children with regard to anthropometric parameters (weight, height, MUAC, head circumference and skin folds). The prevalence of stunting and thinness was similar in both groups. Stunting was 68.3% in the exposed group and 67.3% in the unexposed group (p = 0.90). The prevalence of thinness was 12.8% in the exposed group and 9.6% in the unexposed group (p = 0.57).

**Table 2:** Anthropometry and nutritional status of children.

Variables	Mother exposed to SAM		*Mother not exposed to SAM*		*p*
	n	Mean ± DS or %	n	Mean ± DS or %	
Weight (Kg)	82	19.2 ± 6.3	52	17.4 ± 4.8	0.09
Height (cm)	82	111.3 ± 13.4	52	106.9 ± 12.3	0.057
MUAC (mm)	89	153.9 ± 21.2	50	157.2 ± 25.6	0.42
Cranial perimeter (cm) *Med (Min-Max)*	85	50.0 (45.2–58.2)	53	50.7 (45.5–55.5)	0.48**
Tricipital skin folds (mm) *Med (Min-Max)*	76	1.50 (0.0–4.2)	46	1.45 (0.0–3.3)	0.61**
Edema (%)	86	4.6%	53	1.9%	0.65*
BMI (Kg/m^2^)	82	15.2 ± 1.9	52	14.7 ± 2.6	0.17
BMIAZ	82	‒0.61 ± 1.23	50	‒0.63 ± 1.27	0.90
Thinness (BMIAZ < -2.0)	82	11.0%	50	10.0%	0.86
BMIAZ < -3.0	82	3.7%	50	6.0%	0.67*
HAZ	82	‒2.46 ± 1.36	52	‒2,88 ± 1.62	0.11
Stunting (HAZ < -2.0)	82	68.3%	52	67.3%	0.90
HAZ < -3.0	82	31.7%	52	48.1%	0.05

(**) Mann-Whitney Test. SAM Severe acute malnutrition in childhood

Table 3 shows that the biological profile is almost similar in the two groups if we consider the mean values of glycemia, urea, creatinine and hemogram. No cases of HIV have been observed in the unexposed group while the prevalence is 2.0% in the exposed children. The prevalence of hyperleukocytosis is 10.3% in exposed and 12.2% in unexposed children (p = 0.76). The proportion of children with a creatine level greater than 7.3mg/L is higher in the group of children whose mothers were exposed to SAM in childhood: 23.8% versus 4.2% (p = 0.048). The proportion of children with a GFR > 110 was higher in the exposed group at 41.4% versus 15.8% (p = 0.11). The prevalence of intestinal parasitic infections is 48.9% in the exposed and 61.5% in the unexposed (p = 0.24).

**Table 3:** Biological indicators, figurative elements of the blood, direct stool examination.

Variables	Mother exposed to SAM		*Mother no exposed to SAM*		*p*
	n	Mean ± DS or %	n	Mean ± DS or %	
HIV serology n (%)	86	2.0	58	0	0.535*
**Hemogram**					
White blood cells (*10 ^**3**^ **/ul)**	68	8.543 ± 2.475	41	8.543 ± 2.476	0.58
FL(Lymphocytes)	68	48.7 ± 9.5%	41	49.5 ± 8.4%	0.66
FL(Neutrophils)	44	40.8 ± 10.8%	31	39.8 ± 9.9%	0.70
FL (Others cells)	44	10.9 ± 4.8%	31	10.3 ± 8.1%	0.69
**% Hyperleukocytosis** (WBC > 11000)		10.3%		12.2%	0.76
Red Blood Cells (*10 ^**6**^ **/ul)**	68	4.382 ± 0.455	41	4.300 ± 0.527	0.38
Haemoglobin g/dL	71	12.7 ± 1.2	45	12.2 ± 1.6	0.09
% Haemoglobin <12 g/dL		26.8%		37.8%	0.21
Hématocrit (%)	68	36.3 ± 3.1	41	35.6 ± 3.9	0.30
Platelets (*10 ^**3**^ **/ul)**	68	326.030 ± 102.320	41	347.850 ± 113.581	0.30
% Thrombocytopenia (Plat < 200 000)		8.8%		9.8%	1.00*
**Wintrobe Constant**					
MCV (fL)	68	82.337 ± 4.948	41	82.205 ± 4.551	0.89
MCH (pg/cell)	68	28.716 ± 1.975	41	28.395 ± 1.953	0.41
MCHC (g/dL)	68	34.878 ± 1.052	41	34.566 ± 1.356	0.18
**Biochemistry & CRP**					
Glycemia (mg/dL)	42	99.4 ± 10.0	25	100.5 ± 11.3	0.67
Creatinine (mg/L)	42	6.53 ± 1.35	24	5.99 ± 1.04	0.09
*Creatinine > 8*,*7 mg/L*	3	7,1%	0	*0*.*0%*	0.29*
*Creatinine > 7*,*3 mg/L*	10	23.8%	1	*4*.*2%*	0.046*
GFR (mL/min/1.73m^2^)	29	102.8± 17.7	19	97.5 ± 17.3	0.30
GFR > 110 mL/min (%)		41.4%		15.8%	0.11*
Urea (mg/dL)	42	19.0 ± 7.4	24	20.8 ± 7.8	0.34
CRP—Qualitative (%)	42	11.9	24	20.8	0.33
CRP—Quantitative (mg/dL)	5	63.6 ± 28.8	5	68.2 ± 16.1	0.76
**Intestinal parasites**	47		39		
**Stools with parasites**	23	48.9%	24	61.5%	0.24
*Number of parasites*	23		24		0.51
*One parasites n (%)*	*16*	*69*.*6%*	*19*	*79*.*2%*	
*Two parasites n (%)*	*6*	*26*.*1%*	*5*	*20*.*8%*	
*> = Three parasites n (%)*	*1*	*4*.*3%*	*0*	*0*.*0%*	

(*) Fisher Exact Fisher. SAM Severe acute malnutrition in childhood

MCV = Mean Corpuscular Volume, MCH = Mean Corpuscular Haemoglobin, MCHC = Mean Corpuscular Haemoglobin Concentration). GFR = Glomerular filtration rate. WBC = White blood Cells.

## Discussion

The objective of our study was to assess the nutritional status and profile of commonly used biological indicators of school-aged children whose mothers had been exposed to SAM during childhood. Table 1 shows that the mothers in these two groups have a similar age around 28 years. However, some nutritional indicators such as % of mothers with height < 145 cm and % and MUAC < 230 cm show a statistical difference between the two groups confirming the lean nutritional status of mothers exposed to SAM during childhood. For their children, we observed high prevalence of stunting and thinness in both groups without a statistically significant difference between them (Table 2). With regard to nutritional indicators (Weight, Height, MUAC, Cranial Perimeter, Tricipital Skin Folds and BMI), we did not observe a statistically significant difference between school-aged children born to mothers exposed to SAM during childhood compared to their community controls. However, exposed children had a lower prevalence of anemia compared to the community controls, but without a significant difference (Table 3).

According to the HAZ criteria we observed in both groups a prevalence of stunting of over 65%. This is higher than that described in other African countries such as Madagascar where 34.9% was reported [41], Ghana where 16.2% [42] was reported and Burkina Faso where 8.8% was reported [43]. On the other hand, the prevalence of thinness according to the BMIAZ criteria is close to the values observed in other countries such as Madagascar with 11.2% [41], Ghana with 9.3% [42] and Burkina Faso with 13.7% [43]. However, the prevalence of thinness observed in Miti-Murhesa HZ is higher than that reported in Vietnam among elementary school children 6–9 years, 5.3% out of a larger sample of 2,334 children, whereas the other studies cited above generally had fewer than 600 students [44]. Also, the BMI value observed in our cohort is low compared to that reported in rural South Africa (Dikgale city), where students had an average weight of 16.5 kg/m^2^ for an average age of 9.6 years [45].

Other studies have reported a higher prevalence of thinness, such as in Ethiopia, where a prevalence of 26.7% was observed among 382 students aged 7–13 years [46].

However, a common feature of all these studies is the association between malnutrition in school children and socio-economic indicators related either to the mother or to the family environment in general [42–46].

The prevalence of anemia was lower in the exposed group than in the unexposed group but without a statistically significant difference. However, the prevalence of anemia remains low compared to the rate of 73.0% reported in Ghana [47] and the rate of 40.4% reported in Burkina Faso [43]. We did not observe any differences in the blood count profile between exposed children and their community controls. Similarly, the Winthrop parameters (CVD, HCM and HCMC) had a similar profile in the two groups and were consistent with international reference values [48]. Cases of HIV were observed in the group of children born to mothers exposed to SAM. This is indicative of the frequent association between malnutrition and HIV in sub-Saharan African countries. The HIV rate among malnourished children is 23% in Malawi [49], while a study conducted in the Pediatric ward of the Provincial Hospital of South Kivu shows that HIV is the main infectious cause of death among children hospitalized for malnutrition [50]. However, the two HIV-positive cases in the exposed group did not have thinness or edema. Excluding HIV, the infectious profile of exposed children is not different from that observed in unexposed children based on CRP results, hyperleukocytosis, and prevalence of intestinal parasitosis (Table 3).

Nearly one child in two is infested with intestinal parasites in children born to exposed mothers and many more in the control group (NS). This reflects the precarious environment in which these children live. This prevalence is similar to that reported in elementary school in other sub-Saharan African countries such as Ethiopia, where the prevalence of intestinal parasitic infections was reported to be 48.7% in the Southern Region [51] and 56.7% in the Bahir Region for children with mental disabilities compared to 41.1% among students without disabilities [52]. However, other studies have reported a much lower prevalence of IPI in sub-Saharan African countries. This is the case in a study conducted in elementary school in Accra, Ghana, which reported a prevalence of 15% [53] and another study conducted in Harbou Town, Ethiopia, where the prevalence of IPI was 21.5 percent [54]. The most common type of intestinalis parasites observed in these areas are giardiasis, roundworm, and Entamoeba histolytica. In our cohort, however, Ascaris Lumbricoides and Trichocephalus were more common. No particular prevalence of Giardiasis was observed in either group and schistomiasis was not identified. Indeed, the Miti Murhesa HZ is not an endemic zone for schistomiasis according to the report of the National Program of Neglected Diseases in the DRC [55]. Factors associated with IPIs cited in these studies were family size, lack of latrine services in the household, consumption of raw vegetables, and inability to wear shoes [51].

Biochemically the glycemia, creatinine and urea values were similar in both groups and in line with international reference values [48]. However, the mean creatinine values tended to be higher in children born to mothers exposed to SAM in childhood without reaching statistical significance. Also, the proportion of children who had a creatinine greater than 7.3mg/L was significantly higher in children exposed with a statistically significant difference. The same trend was also observed in subjects who were assessed Glomerular Filtration Rate but without significant difference. Studies have shown that kidney function is reduced in subjects born at low birth weight! But for our cohort we did not have precise information on birth weight [56]. Unfortunately, the size of our sample does not allow us to draw an obvious conclusion but this observation deserves to be further investigated by more methodologically refined studies that specifically explore renal function in the population group exposed to SAM.

Finally, the schooling rate of these children was similar in the two groups (exposed and non-exposed) even if the proportion of children with optimal schooling is higher in the non-exposed children. It should be noted that in the DRC nursery school is not compulsory, and the age required for compulsory education varies between 6–12 years, corresponding to the expected period for elementary school. The proportion of children aged 3–5 years enrolled in kindergarten was estimated at 4.0 percent in 2013 for the country as a whole, but the majority is concentrated in large cities. Nursery school for children under 6 years of age is almost not organized in rural areas [57].

In considering the nutritional status, health and schooling of the children admitted to the study, there were no indicators showing a specific vulnerability of children born to mothers with a history of SAM in childhood compared to other children in the community. This confirms the theory of the primary role of the socio-economic environment in the growth and development of children as supported by Leroy et al. [58].

The strength of our study is to present the nutritional and health status of school-aged children whose parents have survived malnutrition in an environment of endemic malnutrition. Although we observed that this group had similar nutrition and health indicators to other children in the community, this group represents a vulnerable group and should benefit from outreach in accordance with the recommendations of the SUN movement [16]. Also, in a global way, the situation of the child at school age is a good reflection of the child’s family situation and the community context in which the child evolves. Thus, the nutritional and health indicators of the child at this age can make it possible to understand whether society has put in place mechanisms that can prevent the new generation from experiencing nutritional problems as stated in the conceptual framework of malnutrition [59, 60].

While a similar publication for sub-Saharan Africa does not yet exist, our study has a methodological limitation. Firstly, we worked with a sample size minimum while studies on large populations are needed to better detect differences between different groups. Also, the samples for biochemical analyses were limited to a small group of subjects for logistic reasons and the tests are performed on a single blood sample. It is therefore obvious that despite the relevant information that this study reveals, its power remains weak to allow a perfect comparison. The second potential bias is that we have no evidence on the nutritional status of mothers in the unexposed group during childhood. Even though our teams cross-referenced information from the nutrition centers, CHW and chiefs of villages to ensure that the mothers in the unexposed group had never suffered from acute malnutrition in childhood, it is not totally excluded that some of them may have suffered from temporary moderate malnutrition. However, the data collected show that this bias is unlikely as Table 1 shows that the proportion of mothers with a height of less than 145 cm is higher in the exposed group compared to the unexposed group with a statistically significant difference. Finally, the third potential bias is that of survivors. Indeed, a study describing mortality in this cohort shows that the majority of those admitted for severe malnutrition to HPL would die within 5–10 years [59]. It is possible that the survivors had less complicated forms of malnutrition or had benefited early on from socio-economic support that allowed them to grow and live like other members of the community. But despite this methodological limitation, the study makes a particular contribution by describing the situation of children born to mothers who had survived in conditions of extreme nutritional vulnerability compared to members of the same community.

## Conclusion

The results of our research showed that in rural areas of the DRC with endemic malnutrition, school-age children born to mothers with a history of SAM in childhood have similar health-nutrition indicators to those of children born to mothers non exposed to SAM. Further studies on this cohort are needed to follow the evolution of these children and their socio-economic integration in the community.

## Supporting information

S1 File (XLSX)

## Abbreviations

BMI: Body Mass Index
BMIAZ: Body Mass Index for-Age Z- score
CEMUBAC: Centre Médical de l’Université Libre de Bruxelles pour ses Activités de Coopération
CHW: Community Health Worker
CRP: C reactive protein
CRSN: Centre de Recherche en Sciences Naturelles de Lwiro
DRC: Democratic Republic of Congo
GFR: Glomerular Filtration Rate
HIV: Human immunodeficiency virus
HZ: Health zone
LMIC: Low and medium-income countries
LPH: Lwiro Pediatric Hospital
MUAC: Mid-upper arm circumference
NCDs: Noncommunicable diseases
RIPSEC: Renforcement Institutionnel pour les Politiques de Santé basées sur l’Evidence en République Démocratique du Congo
SAM: Severe acute malnutrition
SUN: Scaling Up Nutrition
WHO: World Health Organization
